# Extracellular Modulation of the Silkmoth Sex Pheromone Receptor Activity by Cyclic Nucleotides

**DOI:** 10.1371/journal.pone.0063774

**Published:** 2013-06-03

**Authors:** Tatsuro Nakagawa, Kazushige Touhara

**Affiliations:** 1 Department of Applied Biological Chemistry, Graduate School of Agricultural and Life Sciences, The University of Tokyo, Tokyo, Japan; 2 JST ERATO Touhara Chemosensory Signal Project, Graduate School of Agricultural and Life Sciences, The University of Tokyo, Tokyo, Japan; AgroParisTech, France

## Abstract

Odorants and pheromones are essential to insects as chemical cues for finding food or an appropriate mating partner. These volatile compounds bind to olfactory receptors (Ors) expressed by olfactory sensory neurons. Each insect Or functions as a ligand-gated ion channel and is a heteromeric complex that comprises one type of canonical Or and a highly conserved Orco subunit. Because there are many Or types, insect Ors can recognize with high specificity a myriad of chemical cues. Cyclic nucleotides can modulate the activity of insect Or-Orco complexes; however, the mechanism of action of these nucleotides is under debate. Here, we show that cyclic nucleotides, including cAMP and cGMP, interact with the silkmoth sex pheromone receptor complex, BmOr-1-BmOrco, from the outside of the cell and that these nucleotides act as antagonists at low concentrations and weak agonists at high concentrations. These cyclic nucleotides do not compete with the sex pheromone, bombykol, for binding to the BmOr-1 subunit. ATP and GTP also weakly inhibited BmOr-1-BmOrco activity, but D-ribose had no effect; these findings indicated that the purine moiety was crucial for the inhibition. Only the bombykol receptors have been so far shown to be subject to modulation by nucleotide-related compounds, indicating that this responsiveness to these compounds is not common for all insect Or-Orco complexes.

## Introduction

Olfactory receptors (Ors) play a pivotal role in sensing volatile chemicals in the external environment. Although both vertebrate and invertebrate Ors possess a seven-transmembrane topology, mammalian Ors are G protein-coupled receptors, whereas insect Ors are heteromeric cation channel complexes that are directly gated by chemosignals, including general odorants and pheromones [1], [2], [3]. Each insect Or comprises one type of variable ligand-binding subunit and a highly conserved Orco subunits [4], [5], [6], [7]. For example, *cis*-jasmone, a volatile and a key attractant for the silkworm *Bombyx mori,* is recognized by BmOr-56-BmOrco complex [8], but bombykol, the sex pheromone in silkmoths, is recognized by BmOr-1-BmOrco complex [4]. Although there was a debate as to the mechanism that the Or-Orco complexes function as ion channels [2], [3], [9], [10], several recent studies have shown that both the ligand-selective Or subunit and the Orco co-receptor contributed to ion channel activity [11], [12], [13], [14]. Thus, every type of heteromeric complex exhibits specific, distinct ligand selectivity and distinct channel properties. Given that there are 60–400 potential ligand-binding Or genes in each insect species [8], [15], [16], [17], [18], [19], [20], the insect Or complex represent a large family of ion-channel receptors.

Each type of Or subunit are thought to possess a unique odorant-, pheromone-, or DEET (an insect repellent)-binding site [21], [22], whereas the Orco subunit does not seem to possess ligand-binding activity; this subunit seems to function solely as a co-receptor that transports the complex to dendritic membranes [4], [6], [7]. Recently, however, it has been reported that the Orco itself can form a functional channel that is activated by VUAA1, which is a non-volatile compound that possesses a purine structural motif [12]. These observations indicate that multiple ligand-binding sites exist in each subunit of insect Or complex, and that the channel activity may be regulated by more chemicals than previously thought, including non-volatile compounds other than volatile odorants or pheromones.

Interestingly, cyclic nucleotides seem to participate in Or-mediated signaling in insects. Reportedly, stimulation of insect antennae with an odorant or pheromone *in vivo* causes an elevation of cyclic nucleotides; this finding also indicated that cyclic nucleotides are involved in insect chemosensory signaling [23]. Wicher et al. has proposed that binding of odorants to the canonical Or subunit activates a Gα_s_ pathway that elevates the intracellular cAMP level, and that this elevation in intracellular cAMP modulates channel activity [3], [24]. However, in our previous study, no increase in intracellular cAMP level was observed upon ligand stimulation, and application of GDP-βS, an inhibitor of G-proteins, had no effect on responses of insect Ors [2]. Therefore we concluded that cyclic nucleotides are not involved in the primary insect olfactory signal transduction. This conclusion was supported by the *in vivo* study showing mutant flies lacking G α proteins in OSNs exhibit normal odor-responses [10]. However, we also found that the insect Or-Orco complex was marginally sensitive to two membrane-permeable cyclic nucleotide analogs, 8-Br-cGMP and 8-Br-cAMP, indicating the possibility that cyclic nucleotides can somehow modulate the activity of the Or-Orco complex [2]. Thus, despite the hard efforts, the role of cyclic nucleotides in insect olfactory signal transduction has been not clear.

Here, we attempted to address the question of whether cyclic nucleotides affect insect Or function, and if so, to elucidate the mechanisms of action of these cyclic nucleotides to insect Ors. Rather surprisingly, we found that cyclic nucleotides non-competitively inhibited the response of the BmOr-1-BmOrco complex to bombykol and that these nucleotides acted via the extracellular surface of the plasma membrane. Our findings have intriguing implications for the role of cyclic nucleotides in pheromone detection in the silkmoth *Bombyx mori*.

## Results

### Cyclic Nucleotides Weakly Activate BmOr-1-BmOrco from the Extracellular Side

**Figure 1:**
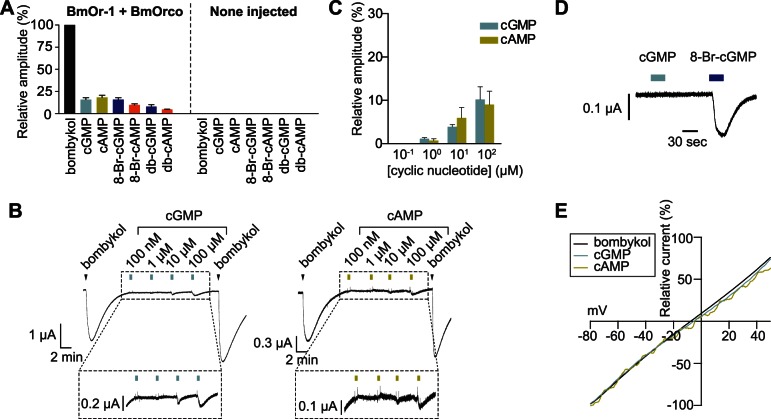
Cyclic nucleotides weakly activate the BmOr-1-BmOrco complex from outside of the cell. (A) Summary of the average responses of BmOr-1-BmOrco-injected oocytes (left) and none-injected oocytes (right) to various cyclic nucleotide reagents (100 µM). Each response was normalized by the response amplitude of BmOr-1-BmOrco-injected oocytes to 10 µM bombykol. The bar graphs show mean ± S.E.M. from 3–4 independent oocytes. (B) Representative current traces of oocytes expressing BmOr-1-BmOrco in response to bombykol and various concentrations of cyclic nucleotides. Bombykol was applied to the oocytes at the time indicated by the arrowhead (for 5 sec), and cyclic nucleotides were applied during the time indicated by colored square (for 30 sec), respectively. Concentrations of ligands were as follows: bombykol, 10 µM, cyclic nucleotides, 100 nM, 1 µM, 10 µM, 100 µM. (C) Dose-dependent responses of BmOr-1-BmOrco to cAMP (yellow) or cGMP (blue). Each point represents the mean current ± S.E.M. from 3 independent oocytes. Each response was normalized to the response to 10 µM bombykol. (D) Representative current traces of oocytes expressing rat olfactory cyclic nucleotide-gated channel complex (CNGA2+A4+B1b) in response to 8-Br-cGMP or cGMP (100 µM). n = 4. (E) Current-voltage relationships of oocytes expressing BmOr-1-BmOrco in response to bombykol (black), cGMP (blue), and cAMP (yellow).

We have previously shown that HEK293T cells expressing BmOr-1-BmOrco or Or47a-Orco were weakly sensitive to extracellular applications of membrane-permeable cyclic nucleotide analogs, 8-Br-cGMP and 8-Br-cAMP [2]. Because these cyclic nucleotide analogs were membrane-permeable, we could not determine whether these reagents acted on the Or-Orco complex via the extracellular or intracellular domain of the complex. To address this question, we applied cGMP, cAMP, db-cGMP, or db-cAMP to *Xenopus laevis* oocytes that expressed BmOr-1-BmOrco. cGMP and cAMP are not membrane-permeable molecules, but db-cGMP and db-cAMP are. The oocytes responded not only to the membrane-permeable cyclic nucleotides, but also to cGMP and cAMP (**Figure. 1A**). None-injected oocytes did not show any response to these reagents (**Figure.1A**). The average amplitudes of BmOr-1-BmOrco expressing oocytes in response to individual cyclic nucleotides (100 µM) were 4.7% to 18% of the amplitudes resulting from responses to bombykol (cGMP, 16±2.1%, cAMP, 18±2.1%, 8-Br-cGMP, 16±2.0%, 8-Br-cAMP, 9.9±1.3%, db-cGMP, 8.0±2.1%, db-cAMP, 4.7±0.4%). To obtain maximal responses, the reagents were applied for 30 sec. The responses were dose-dependent (**Figure. 1B, C**).

To assess the membrane permeability of these reagents, we examined whether oocytes that expressed rat cyclic nucleotide-gated channels (CNGA2+A4+B1b), which has a cyclic nucleotide-binding site only in the intracellular domain [25], were sensitive to these cyclic nucleotide reagents. The oocytes that expressed the CNG channel responded to extracellular application of 8-Br-cGMP, which is membrane permeable, but not to membrane-impermeable cGMP (**Figure. 1D**). These results indicated that the extracellular surface of the BmOr-1-BmOrco complex had a site that interacted with cyclic nucleotides or their analogs.

The current-voltage relationships of cyclic nucleotide-induced responses were similar to that of the bombykol-induced response, indicating that the same ion channel was involved in the response to cyclic nucleotides and to bombykol (**Figure. 1E**). These results indicated that cyclic nucleotides functioned as weak agonists of the bombykol receptor.

**Figure 2:**
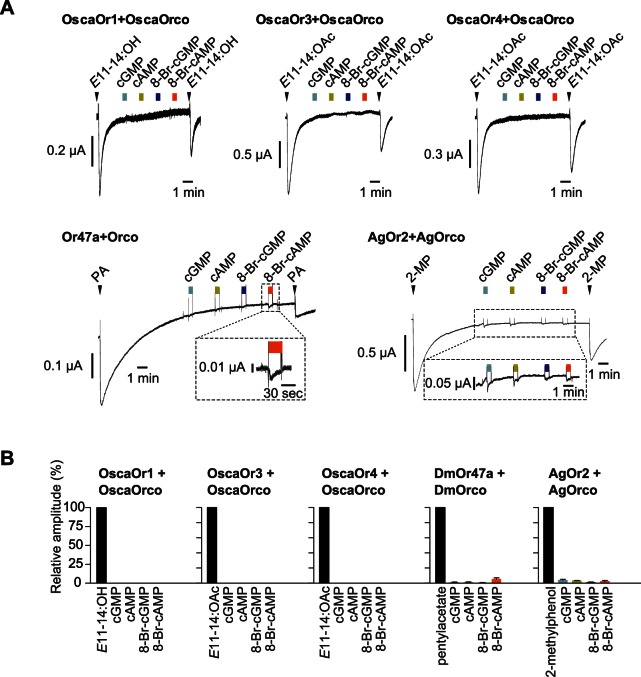
Responsiveness of various Or-Orco complexes to cyclic nucleotides. (A) Representative current traces of oocytes expressing various insect Or-Orco complex in response to their cognate ligands and cyclic nucleotide reagents. Pheromone/odorant was applied to the oocytes at the time indicated by the arrowhead (for 5 sec), and cyclic nucleotide reagent was applied during the time indicated by colored square (for 30 sec), respectively. Concentrations of ligands are as follows: *E*11–14:OH, 10 µM, *E*11–14:OAc, 10 µM, pentylacetate, 100 µM, 2-methylphenol, 100 µM, cyclic nucleotide reagents, 100 µM. (B) Summary of the average responses to cyclic nucleotide reagents. Each response was normalized to a response to the cognate ligand of each Or. The bar graphs show mean ± S.E.M. from 3–4 independent oocytes.

We next examined whether other insect Ors were also sensitive to cyclic nucleotides and their analogs. Specifically, we tested the Or47a-Orco (*Drosophila melanogaster*) and AgOr2-AgOrco (*Anopheles gambiae*) complexes, which represented general odorant receptors [2], and the OscaOr1-OscaOrco, OscaOr3-OscaOrco and OscaOr4-OscaOrco complexes (*Ostrinia scapulalis*), which represented pheromone receptors [26], [27]. We expressed each Or-Orco complex in *Xenopus* oocytes and treated these oocytes with each of the cyclic nucleotides, the two membrane-permeable and the two membrane-impermeable nucleotides. None of Or-Orco complex showed clear responses to any of the nucleotides, but Or47a-Orco and AgOr2-AgOrco each showed a marginal response to each nucleotide (**Figure. 2A, B**). These results indicated that sensitivity to cyclic nucleotides is not a common feature of Or-Orco complexes.

### Cyclic Nucleotides Interact with the BmOr-1 Subunit of the Receptor

**Figure 3:**
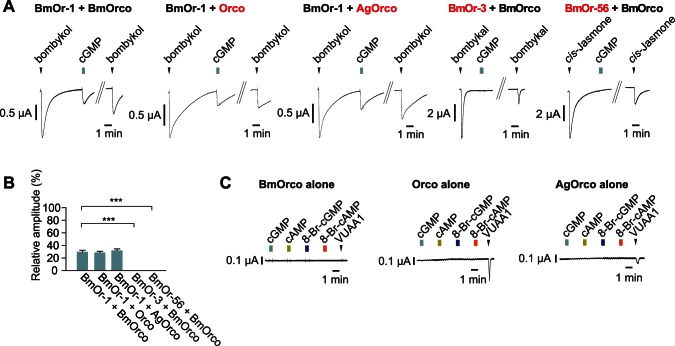
Cyclic nucleotides interact with BmOr-1 subunit of the BmOr-1-BmOrco complex. (A) Representative current traces of oocytes expressing various combinations of the insect Or-Orco complex in response to their cognate ligand or cGMP. Odorant was applied to the oocytes at the time indicated by an arrowhead (for 5 sec), and cGMP was applied during the time indicated by colored square (for 30 sec), respectively. Concentrations of ligands were as follows: bombykol, 10 µM, bombykal, 3 µM, *cis*-Jasmone, 100 µM, cGMP, 100 µM. (B) Summary of the average response amplitudes of the Or-Orco complex to cGMP. The Y-axis is normalized to the response to their cognate ligand. The bar graphs represent mean ± S.E.M. from 3–4 independent oocytes. One-way ANOVA with Tukey’s multiple comparison test, ***p<0.001. (C) Representative current traces of oocytes expressing the Orco family alone in response to cyclic nucleotide reagents or VUAA1 (for 30 sec or 10 sec, respectively). All reagents were applied at the concentration of 100 µM.

We next determined whether the Or subunit or the Orco subunit interacted with the cyclic nucleotides. The cyclic nucleotide sensitivity was dependent on the composition of Or-Orco complex (**Figures. 1** **,** **2** ); therefore, we reasoned that we could determine which subunit was the target of the cyclic nucleotides by changing the subunit composition of the complex. Responses to cGMP were observed in oocytes expressing BmOr-1 and any member of the Orco family (BmOrco, Orco, AgOrco) (**Figure. 3A, B**). On the other hand, oocytes that expressed BmOr-3-BmOrco (bombykal receptor [4]) or BmOr-56-BmOrco did not respond to cGMP (**Figure. 3A, B**). These results indicated that cyclic nucleotide sensitivity was dependent on the canonical Or subunit of the Or-Orco complex. Oocytes that express only an Orco protein did not show any response to cyclic nucleotides, but oocytes injected with any Orco family member but BmOrco responded to VUAA1, which is an allosteric agonist of Orco complexes [12] ( **Figure. 3C**). Taken together, these findings indicated that cyclic nucleotides appeared to interact with the BmOr-1 subunit of the BmOr-1-BmOrco complex.

### Cyclic Nucleotides are Non-competitive Antagonists of BmOr-1-BmOrco

**Figure 4:**
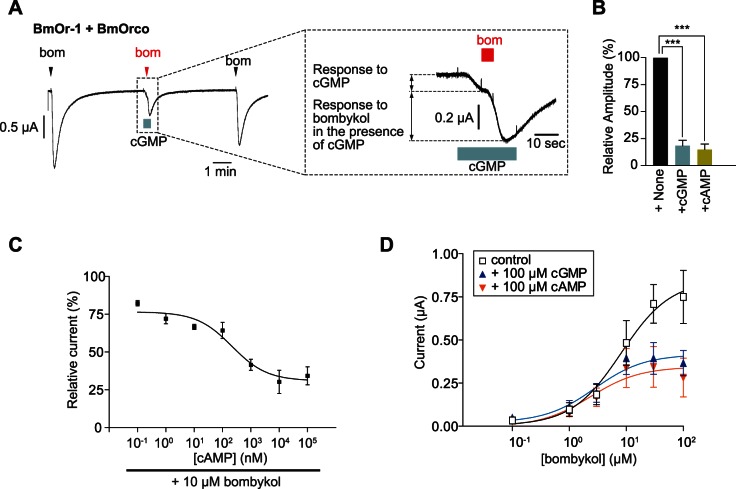
Cyclic nucleotides non-competitively inhibit the response of BmOr-1-BmOrco to bombykol. (A) A representative current trace of an oocyte expressing BmOr-1-BmOrco as it responds to bombykol in the presence of 100 µM cGMP. Bombykol was applied to the oocyte at the time indicated by the arrowhead, and cGMP was applied during the time indicated by light blue square, respectively. The trace for the inhibition of a response to bombykol by cGMP is magnified in an inset figure wherein the response to cGMP is distinguished from that to bombykol in the presence of cGMP. (B) Summary of average responses of BmOr-1-BmOrco to 10 µM bombykol in the absence (+None) or presence (+cGMP, +cAMP) of cyclic nucleotides (100 µM). Each bar represents mean ± S.E.M. from 3–4 independent oocytes. The Y-axis is normalized to the response amplitude of 10 µM bombykol+None condition. Unpaired Student’s t-test, ***p<0.001 vs ‘+None’. (C) Dose-dependent inhibition of BmOr-1-BmOrco by cyclic nucleotides as a percentage of the response to 10 µM bombykol. Each point represents mean ± S.E.M. from 4 independent oocytes. The IC_50_ value is 217 nM. (D) Dose-response curves of responses of BmOr-1-BmOrco to bombykol in the absence or presence of 100 µM cyclic nucleotides. Each point represents mean ± S.E.M. from 4–5 independent oocytes. EC_50_ values are as follows: ‘+None’, 8.2 µM, ‘+cGMP’, 2.8 µM, ‘+cAMP’, 2.7 µM.

In *Bombyx mori*, perfusion of db-cGMP into lymph of pheromone-receptive sensillum caused reduction of an electrical response to bombykol [28]. Therefore, we hypothesized that cyclic nucleotides might inhibit the response of the BmOr-1-BmOrco to bombykol. To assess this hypothesis, we applied bombykol to oocytes that expressed the BmOr-1-BmOrco complex in the presence of a 10-fold amount of cGMP or cAMP compared to that of bombykol. Cyclic nucleotides were applied during and before or after 10 seconds of bombykol application; therefore, we could distinguish between activation and inhibitory effects of the cyclic nucleotides (**Figure. 4A**). Responses of oocytes that expressed BmOr-1-BmOrco to bombykol were significantly reduced in the presence of cGMP or cAMP (**Figure. 4A, B**). The cyclic nucleotides inhibited the response of oocytes to bombykol in a dose-dependent manner; responses in nucleotide-treated oocytes were 10–25% of those in oocytes that were not treated with cyclic nucleotides. The IC_50_ value of the inhibition was 217 nM for cAMP (**Figure. 4C**).

We next sought to determine the mechanism by which nucleotides inhibited BmOr-1-BmOrco. Generally speaking, receptors are subject to one or both of two types of inhibition, competitive or non-competitive inhibition. In the case of competitive inhibition, a dose-response curve is shifted toward higher concentration but there is no change in the maximum response amplitude at the saturated concentration of agonists. In contrast, in the case of non-competitive inhibition, the curve is not shifted, but the maximum response amplitude decreases. In the presence of cyclic nucleotides, the maximum response amplitude of oocytes that expressed BmOr-1-BmOrco was reduced, and there was no change in the EC_50_ value (control: 8.2 µM,+cGMP: 2.8 µM,+cAMP: 2.7 µM) (**Figure. 4D**). These changes were typical of non-competitive inhibition. Taken together, these results indicated that cyclic nucleotides bound to a site within BmOr-1 that was distinct from the bombykol binding site, and that these nucleotides acted as non-competitive antagonists for BmOr-1-BmOrco.

**Figure 5:**
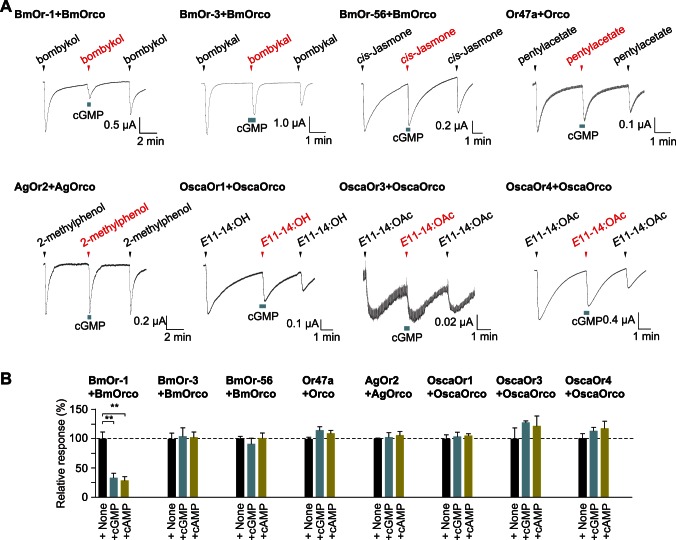
Cyclic nucleotides do not inhibit the responses of Or-Orco types other than BmOr-1-BmOrco. (A) Representative current traces of oocytes that each expressed a different Or-Orco complex; these oocytes were responding to their cognate ligands in the absence or presence of 100 µM cGMP. Odorant (100 µM *cis*-Jasmone, 100 µM pentylacetate, 100 µM 2-methylphenol) or pheromone (1 µM bombykol, 3 µM bombykal, 10 µM *E*11–14:OH, and 1 µM *E*11–14:OAc) was applied to the oocytes at the time indicated by the arrowhead, and cGMP was applied during the time indicated by light blue square. (B) Summary of average responses of Or-Orco to their cognate ligands in the absence (+None) or presence (+cGMP, +cAMP) of cyclic nucleotides (100 µM). Each bar represents mean ± S.E.M. from 3–4 independent oocytes. The Y-axis is normalized to the response amplitude of 10 µM bombykol+None condition. Unpaired Student’s t-test, **p<0.01 vs ‘+None’.

We next examined whether any Or-Orco other than BmOr-1-BmOrco was also inhibited by cyclic nucleotides. We expressed seven different Or-Orco complexes (i.e. BmOr-3-BmOrco, BmOr-56-BmOrco, Or47a-Orco, AgOr2-AgOrco, OscaOr1-OscaOrco, OscaOr3-OscaOrco, or OscaOr4-OscaOrco) individually in oocytes and applied a mixture of cognate odor/pheromone and cyclic nucleotide. None of these seven Or-Orco complexes was inhibited by cyclic nucleotides, indicating that the antagonistic effect of cyclic nucleotide is not a common feature of insect Or-Orcos (**Figure. 5**).

### Structure-activity Relationship of Weak Activation and Inhibition of BmOr-1-BmOrco by Nucleotide-related Compounds

**Figure 6:**
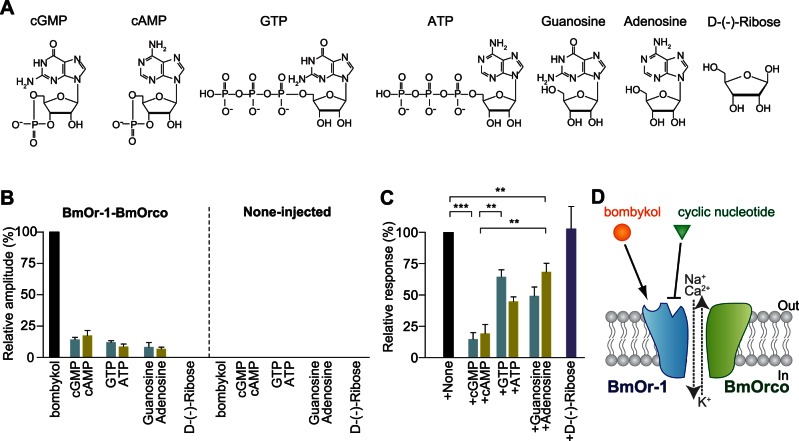
Structure-activity relationship of BmOr-1-BmOrco to cyclic nucleotides and related reagents. (A) Structural formulae of reagents examined in this assay. (B) Summary of the average responses of BmOr-1-BmOrco-injected oocytes (left) and none-injected oocytes (right) to various nucleotide-related reagents (100 µM). Each response was normalized to the response of BmOr-1-BmOrco-injected oocytes to 10 µM bombykol. Each bar represents mean ± S.E.M. from 3 independent oocytes. (C) Summary of inhibition of the response to 10 µM bombykol by various nucleotide-related reagents (100 µM). The Y-axis is normalized to the response to 10 µM bombykol. Each bar represents mean ± S.E.M. from 3 independent oocytes. One-way ANOVA with Tukey’s multiple comparison test, **p<0.01, ***p<0.001. (D) A model showing the mechanism of action of cyclic nucleotides to the bombykol receptor (BmOr-1-BmOrco) complex. Cyclic nucleotides modulate the activity of the complex by binding to the allosteric site in the extracellular domain of the BmOr-1 subunit.

Finally, we examined the structure-activity relationship of the responsiveness of BmOr-1-BmOrco using various nucleotide-related compounds, including two cyclic nucleotides (cGMP and cAMP) and five other structurally related chemicals (GTP, ATP, guanosine, adenosine, and D-(−)-ribose) (**Figure. 6A**). Each compound, other than D-(−)-ribose, exhibited weak agonist activity (**Figure. 6B**) and had an inhibitory effect on the response of BmOr-1-BmOrco to bombykol (**Figure. 6C**). None-injected oocytes did not show any response to those reagents (**Figure. 6B**). The inhibitory effects of GTP, ATP, guanosine, and adenosine were weaker than those of cGMP and cAMP (**Figure. 6C**). These results indicated that the purine structure in the compounds, but not the ribose moiety, was crucial for interaction with BmOr-1-BmOrco complex.

## Discussion

Here, we demonstrated that cyclic nucleotides and related compounds acted as non-competitive inhibitors of the bombykol receptor, BmOr-1-BmOrco. Although this sensitivity to nucleotides appears to be not common for all insect Or-Orco complexes, nucleotide-related compounds may play a role in the modulation of pheromone responsiveness in silkmoths. The binding site(s) for the compounds resides in the BmOr-1 subunit, and it is a rather surprising result that these inhibitory nucleotides interacted with the bombykol receptor on the extracellular surface of the plasma membrane (**Figure. 6D**). Our results support the notion that insect Or channels possess multiple ligand binding sites in the canonical Or subunit and at least one site in the Orco subunit, and therefore, are positively and negatively regulated by multiple factors [12], [29], [30].

Cyclic nucleotides have been proposed to function as intracellular second messengers in insect olfactory signal transduction. *Antheraea polyphemus* OSNs show electrical responses to cGMP from the intracellular side [31]. Furthermore, by using a heterologous expression system, it has been shown that cyclic nucleotides mediate insect olfactory signal transduction by binding to Orco subunit [3], [24]. Electrical responses to 8-Br-cAMP have been observed in whole-cell or inside-out patch membranes of HEK293 cells expressing Orco alone [3], [24]. Contrary to those reports, we showed that cyclic nucleotides bound to and activate the BmOr-1 subunit from the extracellular side and the Orco subunit was not involved in cyclic nucleotide sensitivities. Currently, we do not understand the reason for this apparent contradiction. Nonetheless, our results indicated that cyclic nucleotides have a previously unappreciated role as extracellular factors that modulate the function of the bombykol receptor. Similar phenomena that cAMP and cGMP modulate functions of proteins from outside of the cells are reported in some other biological systems [32], [33], [34]. Thus, it is fair to say that insect Ors are regulated by cyclic nucleotides, but the mode or mechanism of regulation appears to be different between species and between receptors. Although BmOr-1 is so far the sole pheromone receptor that possesses cyclic nucleotides sensitivity, there may be other pheromone receptors that can be modulated by cyclic nucleotides as well. It is of note that several groups have observed the pheromone-dependent rise in cGMP in trichoid sensilla of *Antheraea polyphemus* and *Manduca sexta* [23], [35].

BmOr-1-BmOrco activity was modulated by various nucleotide-related compounds, but not by D-(−)-ribose. Although we could not test a lone purine base due to insolubility of these compounds, the structure-activity relationship study suggested that the purine base was crucial for the binding activity to BmOr-1-BmOrco. A purine consists of a pyrimidine ring fused to an imidazole ring. VUAA1 possess a pyridine ring and a triazoline skeleton [12]. It is interesting to note that both purine and VUAA1 are nitrogen-containing compounds. This might be a common feature for compounds that regulate activity of insect Or complexes.

There are many proteins with functions that are regulated by cyclic nucleotides, including cyclic nucleotide-gated channels, cGMP- and cAMP-dependent protein kinases, and the *E. coli* catabolite gene activator protein (CAP) [36], [37]. These proteins share amino acid sequences that are involved in the binding of cyclic nucleotides [36], [37]. Neither BmOr-1 nor BmOrco possess the cyclic nucleotide binding sequence conserved in the conventional cyclic nucleotide-binding proteins. In this regard, the BmOr-1-BmOrco complex may be a novel type of cyclic nucleotide-binding protein.

Several groups have proposed that cyclic nucleotides are involved in adaptation of olfactory sensory neurons in moths. Perfusion of db-cGMP into sensillum lymph of olfactory sensilla attenuates the bombykol response in *Bombyx mori* [28]. Additionally, stimulation of male silkmoth antennae with bombykol reportedly causes a long-lasting (nearly 30 min) elevation of the cGMP level within either the soma of olfactory sensory neurons or the surrounding auxiliary cells [23]. These cells synthesize and secrete various chemicals and proteins into sensillum lymph [38]; therefore, we hypothesize that cyclic nucleotides (or other nucleotides) are synthesized in the surrounding auxiliary cells and are secreted into lymph after the stimulation of sex pheromones. The activity of pheromone-sensitive OSNs of *Bombyx mori* may be suppressed as a result of inhibition of BmOr-1-BmOrco complex.

Our results suggest that the action of receptor modulation by cyclic nucleotides is different depending on the concentration. At concentrations between 10 nM and 10 µM, cAMP or cGMP acted as antagonists of 10 µM bombykol (IC_50_ = 217 nM), but these compounds function as weak agonists at higher than 10 µM. Unfortunately, it is not possible to know the exact concentration of bombykol in sensillum lymph when male moths are exposed to bombykol emitted from female moths. Furthermore, the time course of increases in the amount of cyclic nucleotides subsequently secreted in the lymph upon pheromone stimulation is unclear. Nonetheless, our results showed reasonable concentration-dependent kinetics such that, at a physiological concentration, cyclic nucleotides may act as an antagonist of the bombykol receptor rather than as an agonist.

We propose the intriguing possibility that the bombykol receptor was attenuated by nucleotide-related compounds that were acting on the extracellular surface of the plasma membrane. In the future, *in vivo* studies will be necessary to assess whether inhibition of the bombykol response that is mediated by these compounds is physiologically relevant. More precisely, the following three questions must be addressed. Does the perfusion of membrane-impermeable cyclic nucleotides into sensillum lymph of adult male *Bombyx mori* suppress the electrical and behavioral responses to bombykol? What is the physiological concentration of cyclic nucleotides in sensillum lymph of *Bombyx mori*? Is the amount of cyclic nucleotides elevated or reduced in some specific physiological timing? Answers to these questions will provide us with information on the meaning and the evolutional significance of the cyclic nucleotide sensitivity of the pheromone receptor in silkmoths.

## Materials and Methods

### Odorants and Pheromones

Bombykol and bombykal were synthesized as previously described [39]. *E*11–14:OAc and *E*11–14:OH were obtained from Pherobank (Wageningen, The Netherlands). VUAA1 was kindly provided by Dr. Leslie Vosshall (Rockefeller Univ.). Stock solutions of these reagents were prepared in DMSO (bombykol and bombykal: 30 mM, *E*11–14:OAc and *E*11–14:OH: 300 mM, VUAA1∶100 mM), and stored at −20°C. These stocks were diluted prior to use in experiments. Pentylacetate and 2-methylphenol were purchased from Tokyo Kasei (Tokyo, Japan) and were directly diluted into a control standard solution (115 mM NaCl, 2.5 mM KCl, 1.8 mM BaCl_2_, and 10 mM HEPES, titrated to pH 7.2 with NaOH) at a final working concentration of 10 or 100 µM. All cyclic nucleotide reagents and related compounds were purchased from Sigma-Aldrich (St. Louis, MO, USA) and were directly diluted into a control standard solution.

### Receptor Expression in *Xenopus laevis* Oocytes and Two-electrode Voltage-clamp Recording

Stage V to VII oocytes were treated with 2 mg/ml of collagenase B (Roche Diagnostics, Tokyo, Japan) in Ca^2+^-free saline solution (82.5 mM NaCl, 2 mM KCl, 1 mM MgCl_2_, and 5 mM HEPES, pH 7.5) for 1 to 2 h at room temperature. Each cRNA was synthesized from a linearized modified pSPUTK vector [40]. For the experiments using insect Or-Orco complexes, oocytes were microinjected with 25 ng of canonical Or cRNA (BmOr-1, BmOr-3, BmOr-56, OscaOr1, OscaOr3, OscaOr4, Or47a, or AgOr2) and 25 ng of the Orco family cRNA (BmOrco, Orco, or AgOrco). For the experiments using oocytes expressing Orco family alone, the oocytes were microinjected with 50 ng of the Orco family cRNA. For the control experiment using rat CNG channel, oocytes were microinjected with 25 ng of CNGA2 cRNA and 12.5 ng of CNGA4 and CNGB1b cRNA. Injected oocytes were incubated for 3–4 days at 18°C in bath solution supplemented with 10 µg/ml of penicillin and streptomycin.

Whole-cell currents were recorded using the two-electrode voltage-clamp technique. Intracellular glass electrodes were filled with 3 M KCl. Signals were amplified with an OC-725C amplifier (Warner Instruments, Hamden, CT, USA), low-pass filtered at 50 Hz and digitized at 1 kHz. The control standard solution contained 115 mM NaCl, 2.5 mM KCl, 1.8 mM BaCl_2_, and 10 mM HEPES, and titrated to pH 7.2 with NaOH. For the control that involved the rat CNG channel, BaCl_2_ was removed from the solution, because divalent cations inhibit the response of CNG channels [41].

The current-voltage relationship was measured by changing membrane potential from −80 mV to +50 mV using voltage-ramp and analyzed by IgorPro software (WaveMetrics, Portland, OR, USA). Ligands were delivered through the superfusing bath solution via a silicon tube that was connected to a computer-driven solenoid valve. Data acquisition and analysis were carried out with Digidata1322A (Axon instruments, Foster city, CA, USA) and pCLAMP software (Axon instruments, Foster city, CA, USA).

### Calculation of Agonist or Antagonist Activity of Cyclic Nucleotides

Agonistic activities of cyclic nucleotides (Figures. 1A, 1D, 2B, 3B, 6B) were calculated as relative amplitude compared to the amplitude in response to first application of a cognate ligand of each pheromone/olfactory receptor in the same oocytes. Antagonistic activities of cyclic nucleotides (responses to cognate ligands in the presence of cyclic nucleotides that are shown in Figures. 4B, 5B, 6C) were calculated as relative amplitude compared to the response amplitude to cognate ligands in the absence of cyclic nucleotides. To avoid the overestimation of inhibitory effect due to desensitization of odor responses by repeated odor application,+None and +[cyclic nucleotide] were respectively calculated from independent ooyctes.

## References

[1] Touhara K, Vosshall LB (2009) Sensing odorants and pheromones with chemosensory receptors. Annu Rev Physiol 71: 307–332. 19575682 10.1146/annurev.physiol.010908.163209

[2] Sato K, Pellegrino M, Nakagawa T, Nakagawa T, Vosshall LB, et al (2008) Insect olfactory receptors are heteromeric ligand-gated ion channels. Nature 452: 1002–1006. 18408712 10.1038/nature06850

[3] Wicher D, Schafer R, Bauernfeind R, Stensmyr MC, Heller R, et al (2008) *Drosophila* odorant receptors are both ligand-gated and cyclic-nucleotide-activated cation channels. Nature 452: 1007–1011. 18408711 10.1038/nature06861

[4] Nakagawa T, Sakurai T, Nishioka T, Touhara K (2005) Insect sex-pheromone signals mediated by specific combinations of olfactory receptors. Science 307: 1638–1642. 15692016 10.1126/science.1106267

[5] Neuhaus EM, Gisselmann G, Zhang W, Dooley R, Stortkuhl K, et al (2005) Odorant receptor heterodimerization in the olfactory system of *Drosophila melanogaster* . Nat Neurosci 8: 15–17. 15592462 10.1038/nn1371

[6] Benton R, Sachse S, Michnick SW, Vosshall LB (2006) Atypical membrane topology and heteromeric function of *Drosophila* odorant receptors in vivo. PLoS Biol 4: e20. 16402857 10.1371/journal.pbio.0040020 PMC1334387

[7] Larsson MC, Domingos AI, Jones WD, Chiappe ME, Amrein H, et al (2004) *Or83b* encodes a broadly expressed odorant receptor essential for *Drosophila* olfaction. Neuron 43: 703–714. 15339651 10.1016/j.neuron.2004.08.019

[8] Tanaka K, Uda Y, Ono Y, Nakagawa T, Suwa M, et al (2009) Highly selective tuning of a silkworm olfactory receptor to a key mulberry leaf volatile. Curr Biol 19: 881–890. 19427209 10.1016/j.cub.2009.04.035

[9] Smart R, Kiely A, Beale M, Vargas E, Carraher C, et al (2008) *Drosophila* odorant receptors are novel seven transmembrane domain proteins that can signal independently of heterotrimeric G proteins. Insect Biochem Mol Biol 38: 770–780. 18625400 10.1016/j.ibmb.2008.05.002

[10] Yao CA, Carlson JR (2010) Role of G-proteins in odor-sensing and CO2-sensing neurons in *Drosophila* . J Neurosci 30: 4562–4572. 20357107 10.1523/JNEUROSCI.6357-09.2010 PMC2858456

[11] Nichols AS, Chen S, Luetje CW (2011) Subunit Contributions to Insect Olfactory Receptor Function: Channel Block and Odorant Recognition. Chem Senses 36: 781–790. 21677030 10.1093/chemse/bjr053 PMC3195787

[12] Jones PL, Pask GM, Rinker DC, Zwiebel LJ (2011) Functional agonism of insect odorant receptor ion channels. Proc Natl Acad Sci U S A 108: 8821–8825. 21555561 10.1073/pnas.1102425108 PMC3102409

[13] Pask GM, Jones PL, Rutzler M, Rinker DC, Zwiebel LJ (2011) Heteromeric anopheline odorant receptors exhibit distinct channel properties. PLoS One 6: e28774. 22174894 10.1371/journal.pone.0028774 PMC3235152

[14] Nakagawa T, Pellegrino M, Sato K, Vosshall LB, Touhara K (2012) Amino acid residues contributing to function of the heteromeric insect olfactory receptor complex. PLoS One 7: e32372. 22403649 10.1371/journal.pone.0032372 PMC3293798

[15] Scott K, Brady RJ, Cravchik A, Morozov P, Rzhetsky A, et al (2001) A chemosensory gene family encoding candidate gustatory and olfactory receptors in *Drosophila* . Cell 104: 661–673. 11257221 10.1016/s0092-8674(01)00263-x

[16] Hill CA, Fox AN, Pitts RJ, Kent LB, Tan PL, et al (2002) G protein-coupled receptors in *Anopheles gambiae* . Science 298: 176–178. 12364795 10.1126/science.1076196

[17] Fox AN, Pitts RJ, Robertson HM, Carlson JR, Zwiebel LJ (2001) Candidate odorant receptors from the malaria vector mosquito *Anopheles gambiae* and evidence of down-regulation in response to blood feeding. Proc Natl Acad Sci U S A 98: 14693–14697. 11724964 10.1073/pnas.261432998 PMC64743

[18] Robertson HM, Wanner KW (2006) The chemoreceptor superfamily in the honey bee, *Apis mellifera*: expansion of the odorant, but not gustatory, receptor family. Genome Res 16: 1395–1403. 17065611 10.1101/gr.5057506 PMC1626641

[19] Wanner KW, Anderson AR, Trowell SC, Theilmann DA, Robertson HM, et al (2007) Female-biased expression of odourant receptor genes in the adult antennae of the silkworm, *Bombyx mori* . Insect Mol Biol 16: 107–119. 17257213 10.1111/j.1365-2583.2007.00708.x

[20] Engsontia P, Sanderson AP, Cobb M, Walden KK, Robertson HM, et al (2008) The red flour beetle’s large nose: an expanded odorant receptor gene family in *Tribolium castaneum* . Insect Biochem Mol Biol 38: 387–397. 18342245 10.1016/j.ibmb.2007.10.005

[21] Nichols AS, Luetje CW (2010) Transmembrane segment 3 of *Drosophila melanogaster* odorant receptor subunit 85b contributes to ligand-receptor interactions. J Biol Chem 285: 11854–11862. 20147286 10.1074/jbc.M109.058321 PMC2852922

[22] Pellegrino M, Steinbach N, Stensmyr MC, Hansson BS, Vosshall LB (2011) A natural polymorphism alters odour and DEET sensitivity in an insect odorant receptor. Nature 478: 511–514. 21937991 10.1038/nature10438 PMC3203342

[23] Ziegelberger G, van den Berg MJ, Kaissling KE, Klumpp S, Shultz JE (1990) Cyclic GMP levels and guanylate cyclase activity in pheromone-sensitive antennae of the silkmoths *Antheraea polyphemus* and *Bombyx mori* . J Neurosci 10: 1217–1225. 1970356 10.1523/JNEUROSCI.10-04-01217.1990 PMC6570213

[24] Sargsyan V, Getahun MN, Llanos SL, Olsson SB, Hansson BS, et al (2011) Phosphorylation via PKC Regulates the Function of the *Drosophila* Odorant Co-Receptor. Front Cell Neurosci 5: 5. 21720521 10.3389/fncel.2011.00005 PMC3118453

[25] Varnum MD, Black KD, Zaqotta WN (1995) Molecular mechanism for ligand discrimination of cyclic nucleotide-gated channels. Neuron 15: 619–625. 7546741 10.1016/0896-6273(95)90150-7

[26] Miura N, Nakagawa T, Tatsuki S, Touhara K, Ishikawa Y (2009) A male-specific odorant receptor conserved through the evolution of sex pheromones in *Ostrinia* moth species. Int J Biol Sci 5: 319–330. 19421342 10.7150/ijbs.5.319 PMC2677733

[27] Miura N, Nakagawa T, Touhara K, Ishikawa Y (2010) Broadly and narrowly tuned odorant receptors are involved in female sex pheromone reception in *Ostrinia* moths. Insect Biochem Mol Biol 40: 64–73. 20044000 10.1016/j.ibmb.2009.12.011

[28] Redkozubov A (2000) Guanosine 3′,5′-cyclic monophosphate reduces the response of the Moth’s olfactory receptor neuron to pheromone. Chem Senses 25: 381–385. 10944500 10.1093/chemse/25.4.381

[29] Jones PL, Pask GM, Romaine IM, Taylor RW, Reid PR, et al (2012) Allosteric antagonism of insect odorant receptor ion channels. PLos One 7: e30304. 22272331 10.1371/journal.pone.0030304 PMC3260273

[30] Chen S, Luetje CW (2012) Identification of new agonists and antagonists of the insect odorant receptor co-receptor subunit. PLoS One 7: e36784. 22590607 10.1371/journal.pone.0036784 PMC3348135

[31] Zufall F, Hatt H (1991) Dual activation of a sex pheromone-dependent ion channel from insect olfactory dendrites by protein kinase C activators and cyclic GMP. Proc Natl Acad Sci U S A 88: 8520–8524. 1717980 10.1073/pnas.88.19.8520 PMC52540

[32] Cervetto C, Maura G, Marcoli M (2010) Inhibition of presynaptic release-facilitatory kainate autoreceptors by extracellular cyclic GMP. J Pharmacol Exp Ther 332: 210–219. 19794031 10.1124/jpet.109.154955

[33] Hofer AM, Lefkimmiatis K (2007) Extracellular calcium and cAMP: second messengers as “third messengers”? Physiology 22: 320–327. 17928545 10.1152/physiol.00019.2007

[34] Nascimento NR, Kemp BA, Howell NL, Gildea JJ, Santos CF, et al (2011) Role of SRC family kinase in extracellular renal cyclic guanosine 3′,5′-monophosphate- and pressure-induced natriuresis. Hypertension 58: 107–113. 21482955 10.1161/HYPERTENSIONAHA.110.168708 PMC3117057

[35] Stengl M, Zintl R, De Vente J, Nighorn A (2001) Localization of cGMP immunoreactivity and of soluble guanylyl cyclase in antennal sensilla of the hawkmoth *Manduca sexta* . Cell Tissue Res 304: 409–421. 11456418 10.1007/s004410000336

[36] Kaupp UB, Niidome T, Tanabe T, Terada S, Bonigk W, et al (1989) Primary structure and functional expression from complementary DNA of the rod photoreceptor cyclic GMP-gated channel. Nature 342: 762–766. 2481236 10.1038/342762a0

[37] Shabb JB, Corbin JD (1992) Cyclic nucleotide-binding domains in proteins having diverse functions. J Biol Chem 267: 5723–5726. 1313416

[38] Gnatzy W, Weber KM (1978) Tormogen cell and receptor-lymph space in insect olfactory sensilla. Fine structure and histochemical properties in *Calliphora* . Cell Tissue Res 189: 549–554. 148970 10.1007/BF00209140

[39] Sakurai T, Nakagawa T, Mitsuno H, Mori H, Endo Y, et al (2004) Identification and functional characterization of a sex pheromone receptor in the silkmoth *Bombyx mori* . Proc Natl Acad Sci U S A 101: 16653–16658. 15545611 10.1073/pnas.0407596101 PMC528734

[40] Katada S, Nakagawa T, Kataoka H, Touhara K (2003) Odorant response assays for a heterologously expressed olfactory receptor. Biochem Biophys Res Commun 305: 964–969. 12767924 10.1016/s0006-291x(03)00863-5

[41] Park CS, MacKinnon R (1995) Divalent cation selectivity in a cyclic nucleotide-gated ion channel. Biochemistry 34: 13328–13333. 7577917 10.1021/bi00041a008

